# Clinical features and risk factors for Sjogren’s syndrome patients suffering from oral candidiasis in Shanxi, China

**DOI:** 10.1186/s12903-024-04595-x

**Published:** 2024-07-17

**Authors:** Yexing Xing, Honghong Shi, Caihong Wang, Ying Yang

**Affiliations:** The Second Hospital of Shanxi Medical University, Taiyuan, Shanxi Province China

**Keywords:** Oral candidiasis, Sjögren’s syndrome, Oral lesions, *Candida* albicans

## Abstract

**Objectives:**

To investigate the clinical features and risk factors of Sjogren’s Syndrome (SS) patients suffering from oral candidiasis and to provide a foundation for the prevention and treatment of oral candidiasis in SS patients.

**Methods:**

The medical records of 479 SS patients admitted to the Second Hospital of Shanxi Medical University from 2018 to 2020 were analysed to determine the clinical characteristics and risk factors that influence the occurrence of oral candidiasis infection in SS patients.

**Results:**

Patients with oral candidiasis were older than those without oral candidiasis (*P* < 0.05). Male SS patients had greater oral candidiasis rates (*P* < 0.05). Unstimulated whole saliva (UWS) and stimulated whole saliva (SWS) were both shown to be adversely associated with oral *Candida* infections (*P* < 0.001). Logistic regression revealed that a low UWS was an independent risk factor for oral *Candida* infections in SS patients (OR: 0.004, *P* = 0.023). Greater WBC counts (OR: 1.22, *P* < 0.001), lower haemoglobin levels (OR: 0.97, *P* = 0.007), lower serum albumin levels (OR: 0.88, *P* < 0.001), lower IgG levels (OR: 0.91, *P* = 0.011), lower IgA levels (OR: 0.75, *P* = 0.011), and lower IgM levels (OR: 0.91, *P* = 0.015) were found in patients with oral *Candida* infections. Patients on immunosuppressive medications (OR: 0.32, *P* = 0.011), particularly rapamycin (*P* < 0.001), had a decreased rate of oral *Candida* infections.

**Conclusions:**

Patients with oral candidiasis were older than those without oral candidiasis. Male SS patients are more likely to have oral candidiasis. Individuals with lower UWS and SWS are more susceptible to oral *Candida* infection. Oral *Candida* infections in SS patients depend on their immunological status. Rapamycin may increase the abundance of Treg cells to reduce oral *Candida* infection in SS patients.

**Supplementary Information:**

The online version contains supplementary material available at 10.1186/s12903-024-04595-x.

## Introduction

Sjögren syndrome (SS) is a systemic autoimmune exogenous disease characterized by dry mouth and eyes, weariness, joint discomfort, and other symptoms [1]. Oral symptoms of SS include xerostomia and hyposalivation, dental caries, oral candidiasis, dental erosion and/or abrasion, and others [2]. Hyposalivation might delay the clearance of sugar and acid from the dental surface [3]. Reduced salivary buffer capacity may lead to oral bacteria and fungi proliferation, resulting in tooth cavities, erosion, candidiasis, and other problems [4].

*Candida* is a commensal yeast found in the oral flora of healthy people. However, its frequency among SS patients is thought to be greater [5, 6]. For SS patients lacking saliva-mediated prevention of Candida adhesion to the mucosa [7], oral signs of candidiasis were evident in 13.1–60% of SS patients [8, 9], which was significantly greater than the rate of candidiasis in healthy controls [10, 11]. In one study, 87.5% of individuals with hyposalivation had *Candida* in their saliva, the majority (80.6%) of which were *Candida* albicans [12]. The aetiology is primarily based on the fact that in SS patients, salivary gland hypoplasia reduces the concentration of immunoglobulins and other electrolytes [13], which increases mucosal exposure to oral flora, particularly *Candida* species [14].

Although the pathogenesis of SS is unclear, it is most likely related to epithelial dysregulation, which attracts immune cells. All epithelial tissues are vulnerable, but the salivary and lacrimal glands are especially vulnerable [15]. The salivary glands are generally sterile environments with strictly controlled immune responses. In addition to IgA-producing plasma cells, salivary gland tissue contains macrophages, dendritic cells, innate lymphoid cells, and T cells, all of which contribute to the defence of oral tissues. Increased infiltration and hyperactivity of these immune cell populations contribute to the development of illnesses such as SS [16]. Studies have focused on salivary decreases and the incidence of oral *Candida* infections in SS patients. Moreover, it has been demonstrated that infections might activate autoimmunity and initiate the pathogenesis of SS [17]. Therefore, we hypothesised that oral *Candida* infections in SS patients are related to the patient’s autoimmune status and that oral *Candida* infections may be associated with widespread activation of immune pathways, leading to chronic dysregulation of T lymphocytes and B lymphocytes.

In this study, we first thoroughly studied the clinical characteristics and risk factors for patients with oral *Candida* infection. Second, we examined the differences in B- and T-cell populations, immunoglobulins, and inflammatory markers between persons infected with SS oral *Candida* and those who were not. The aim of this study was to understand the relationship between patients’ autoimmune levels and oral *Candida* infections to help predict the development of oral *Candida* infections in SS patients, providing a theoretical foundation for early clinical prevention and therapy.

## Materials and methods

### Participants

This was a single-centre, retrospective observational study of Sjögren’s syndrome patients admitted to the Rheumatism and Immunology Department at Shanxi Medical University’s Second Hospital in China. This research included 479 Sjögren’s syndrome patients from the Department of Rheumatology and Immunology at Shanxi Medical University’s Second Hospital in China from 2018 to 2020. All participants who had not received antibiotics in the preceding 3 months and had no systemic diseases (including hepatitis, tuberculosis, and diabetes) and no smoking or alcohol abuse were enrolled. None of the 479 patients presented secondary SS. If selected patients had any other connective tissue disease were excluded. A normal clinical procedure was used, and the following characteristics were recorded: age, sex, medical history, and type and quantity of medications. All experimental protocols were approved by the Human Ethics Review Committee at the Second Hospital of Shanxi Medical University. Informed consent was obtained from all subjects and/or their legal guardian(s).

### SS disease

The individuals were diagnosed with primary SS according to the 2016 ACR-EULAR Classification Criteria for Primary Sjögren’s Syndrome, with a total score ≥ 4 [18]. The classification criteria were based on the weighted sum of 5 items: anti-SSA antibody positivity and focal lymphocytic sialadenitis with a focus score ≥ 1 foci/mm2, each scoring 3; an abnormal ocular staining score ≥ 5 (or van Bijsterveld score ≥ 4); a Schirmer test ≤ 5 mm/5 min; and an unstimulated salivary flow rate ≤ 0.1 mL/min, each scoring 1. Individuals (with signs/symptoms suggestive of SS) who had a total score ≥ 4 for the items above met the criteria for SS.

### Oral symptoms

A calibrated expert in stomatology (JS) performed the full face and intraoral mucosal examinations in our hospital dental clinic during their hospitalization. Individuals diagnosed with oral candidiasis had at least one clinical manifestation and one positive pathogenetic test. Clinical presentation of the primary forms of oral candidiasis includes acute pseudomembranous candidiasis, chronic erythematous candidiasis, acute erythematous candidiasis, and chronic hyperplastic candidiasis. Secondary forms of oral candidiasis can also occur and are frequently described as *Candida*-associated lesions, including angular cheilitis, median rhomboid glossitis [19]. The criteria for positive pathogenetic results included a direct smear showing pseudohyphae or positive fluorescence staining, a positive cotton swab culture and a positive saliva culture with *Candida* counts > 100 colony-forming units (CFU)/ml [20].

### Laboratory examination

For all patients, white blood cells, lymphocytes, platelets, liver and kidney function markers, immunoglobulin series, immunological function markers, T-cell subsets, cytokines, glucocorticoid and immunosuppressive medication, salivary flow rate, and drug sensitivity of oral secretions were assessed. An automated analyser was used to measure the white blood cells, lymphocytes, platelets and liver and kidney function markers in the blood. The immunoglobulin series was detected using an enzyme-linked immunosorbent adsorption assay.

### Saliva sampling

One oral medicine expert (LR) collected both stimulated whole saliva (SWS) and unstimulated whole saliva (UWS). Patients were instructed not to eat, drink, smoke, or clean their teeth for 90 min before the session. The samples were collected between 8:00 and 10:00 a.m., with the UWS sample always collected first for 15 min. Patients were instructed to chew a paraffin gum and spit their saliva into a plastic container for 10 min to obtain the SWS sample. Flow rates were measured in millilitres per minute. A flow rate of 1.0 mL/min for SWS and 0.1 mL/min for UWS was characterised as hyposalivation.

### Flow cytometry analysis

#### Analysis of lymphocyte subsets

Peripheral blood samples (2 mL) from each patient were obtained to measure the percentages and numbers of T, B, CD4, CD8, and NK cells (CD3-CD16 + CD56+). TruCount tubes and Tube B contained 50 µl of each blood sample for immunofluorescence staining. Then, 20µL of CD3-FITC/CD8-phycoerythrin (PE)/CD45-peridinin-chlorophyll protein (PerCP)/CD4-allophycocyanin (APC) antibodies (clones SK7/SK1/2D1/SK3) were added to Tube A, and 20 µL of CD3-FITC/CD16 + 56-PE/CD45-PerCP/CD19-APC antibodies (clones SK7/B73.1 NCAM16.2/2D1/SJ25C1) were added to Tube B. All antibodies were obtained from BD Biosciences (San Jose, CA). After the cells were stained at room temperature for 20 min in the dark, they were washed with 1× FACS buffer and incubated for 15 min in the dark. A BD Bioscience FACSCanto instrument was used to collect 15,000 cell data points for MultiSET software analysis [21].

#### Analysis of CD4 + T cell subsets

To analyse Th1, Th2, and Th17 cells, 80 µL of heparinized blood was stimulated with 10 µL of phorbol myristate acetate, 10 µL of ionomycin and 1 µL of GolgiStop. The cells were cultured at 37 °C for 5 h before being split into tubes A and B. After incubating both tubes with human anti-CD4-FITC (clone SK3) at room temperature for 30 min, the cells were fixed and permeabilized at 4 °C for 30 min. After that, the Tube A cells were incubated with anti-IL-17-PE (clone SCPL1362) and anti-IFN-APC (clone 4 S. B3), and Tube B cells were incubated with human anti-IL-4 PE (clone MP4-25D2) in the dark at room temperature for 30 min. Four-colour flow cytometry was performed on phosphate-buffered saline-washed cells. At room temperature in the dark for 30 min, 80 µl of anticoagulated blood was incubated with anti-CD4-FITC and anti-CD25-APC (clone 2A3) to analyse Treg cells. After 30 min of fixation and permeabilization at 4 °C, the cells were incubated with human anti-FOXP3-PE (clone PCH101) at room temperature for 30 min. After washing, the cells were analysed with a FACSCalibur (BD Biosciences). To calculate CD4 + T-cell subset frequencies, 10,000 cells were gated and analysed using Cell Quest software. Multiplying the proportion of each subgroup by the overall CD4 + T-cell count yielded absolute numbers of subsets. Cell types were defined as Th1 (CD4 + IFN-c+), Th2 (CD4 + IL-4+), Th17 (CD4 + IL-17+), or Treg (CD4 + CD25 + FOXP3+) [21].

### Statistical analysis

SPSS 23.0 was used to evaluate all of the data. The standard deviation (SD) of quantitative variables with a normal distribution was used to describe them, whereas the median (interquartile range) of variables with a skewed distribution was used to describe them. The percentages were used to characterise the categorical variables. If the data were regularly distributed, the t test was used to assess differences in quantitative variables; otherwise, the Mann‒Whitney U test was used. The chi-squared test or Fisher’s exact test was employed to examine categorical variable differences. A P value < 0.05 was considered to indicate statistical significance. For two-by-two comparisons between multiple groups, the chi-squared test or Fisher’s exact test was used, and a P value < 0.017 was considered to indicate statistical significance.

## Results

### Characteristics of patients

This study included 479 Sjogren’s syndrome patients, as shown in Table 1 and S1. The patient ages ranged from 18 to 85 years, with a median of 56 (50–64) years. A total of 447 (93.30%) of the patients were female. The hospital stays ranged from 3 to 42 days, with a median of 13 (10–16) days. Oral candidiasis was observed in 72 (15.03%) of the patients. Glucocorticoids were administered to 357 (74.53%) of the patients. Immunosuppressive medication, comprising rapamycin, hydroxychloroquine, and total glucosides of paeony, was administered to 350 patients (73.07%). complicated.

**Table 1:** General characteristics of patients

	Median (interquartile range) or Percentage
Age(years)	56.00(50.00–64.00)
Male(%)	6.70%
Length of stay in hospital (day)	13.00(10–16)
With oral candidiasis(%)	15.03%
Glucocorticoid treatment(%)	74.53%
Immunosuppressant treatment(%)	73.07%

### Comparisons of patients with and without oral candidiasis

**Fig. 1:**
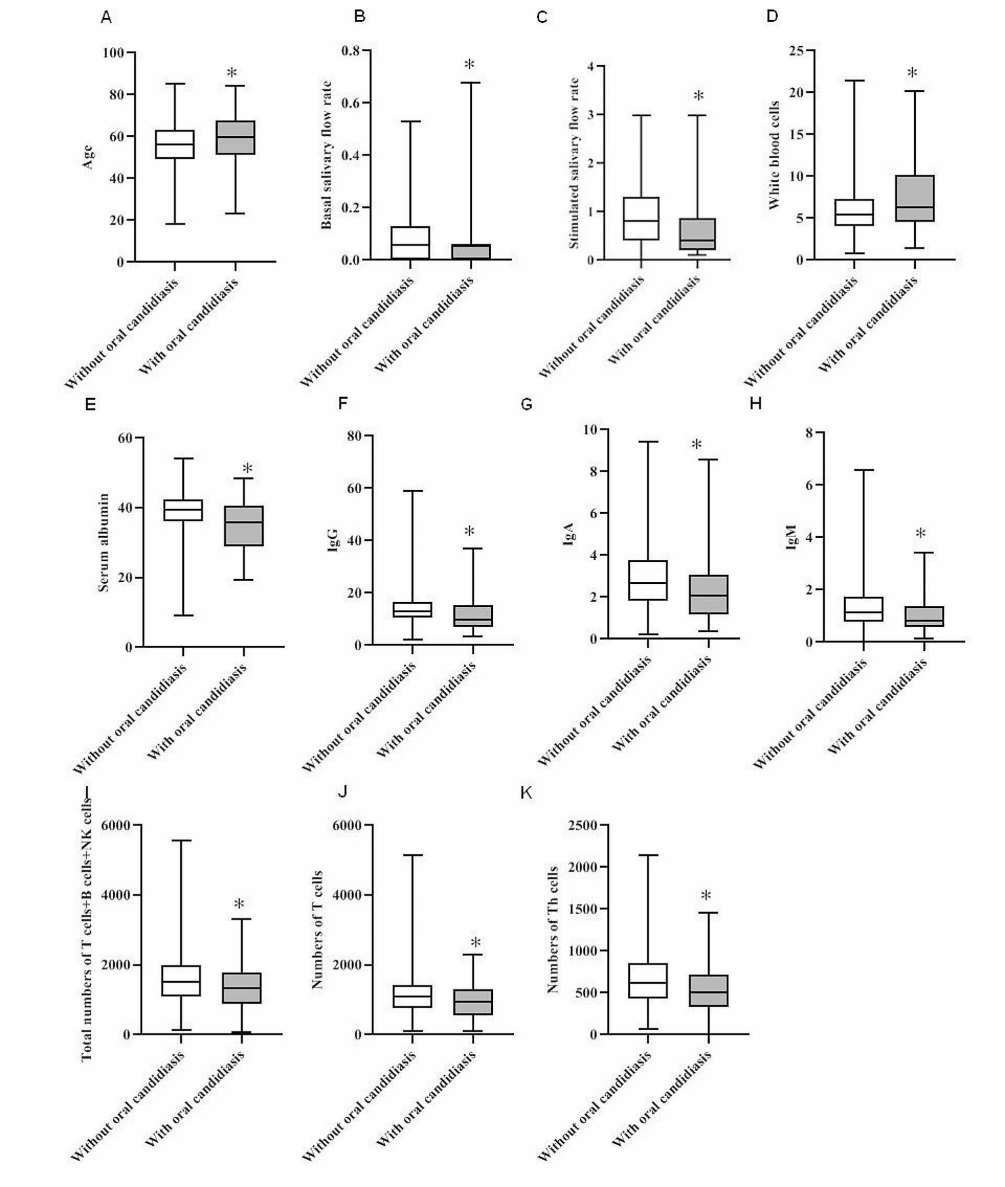
Significant differences in general clinical characteristics between patients with and without oral candidiasis. *P<0.05

The results of comparing patients with oral candidiasis to those without oral candidiasis, as well as the clinical characteristics of all 479 eligible patients included in the research, are displayed in Tables 2 and 3; Fig. 1. Patients with oral candidiasis were older than those without oral candidiasis (*P* < 0.05). Compared with female patients, male SS patients had a greater incidence of oral *Candida* infections (*P* < 0.05). Patients with oral candidiasis had reduced UWS and SWS (*P* < 0.001). White blood cell (WBC) counts were substantially greater in oral candidiasis patients than in control individuals (*P* < 0.01). Serum ALB, IgG, IgA, and IgM levels, the total number of T cells, B cells, and NK cells, and the number of Th cells were considerably lower in individuals with oral candidiasis than in those without oral candidiasis (*P* < 0.05). Patients receiving immunosuppressive medications had less oral candidiasis (*P* < 0.01). A chi-square test was conducted for the groups that received various immunosuppressive medications (*P* < 0.01). Two-by two comparisons were made among the three treatments, namely, rapamycin, hydroxychloroquine, and total white peony glycosides (Table 4). Compared with patients treated with hydroxychloroquine and total glucosides of peony capsules, patients treated with rapamycin had a lower rate of oral candidiasis infection.

**Table 2:** Differences in general clinical characteristics between patients with and without oral candidiasis

	Patients without oral candidiasis	Patients with oral candidiasis	*P*
Age(years)	56.00(49.00–63.00)	59.50(51.25–67.75)	0.022*
Male(%)	5.16%	15.28%	0.004*
Length of stay in hospital (day)	13.00(10–16)	14(10.00–17.00)	0.157
Basal salivary flow rate(mL/min)	0.06(0.00-0.13)	0.06(0.00-0.06)	<0.001*
Stimulated salivary flow rate(mL/min)	0.80(0.40–1.30)	0.40(0.20–0.88)	<0.001*
White blood cells(×10^9^/L)	5.40(4.10–7.25)	6.34(4.52–10.13)	0.003*
Hemoglobin(g/L)	125(116–135)	123(108–134)	0.296
Lymphocyte(×10^9^/L)	1.58(1.15–2.05)	1.49(1.07–2.04)	0.408
Platelets(×10^9^/L)	207.00(156.00-240.00)	215.00(173.00-234.00)	0.496
Alanine transaminase(U/L)	17.50(13.00-25.35)	22.15(13.35–28.60)	0.109
Aspertate aminotransferase(U/L)	21.30(16.80–26.50)	23.30(18.50–28.40)	0.092
Total protein(g/L)	71.30(65.95–75.80)	69.10(62.30-74.95)	0.052
Serum albumin(g/L)	39.40(36.30–42.40)	35.90(28.93–40.53)	<0.001*
Serum urea(mmol/l)	4.90(4.20-6.00)	5.15(4.00-6.45)	0.611
Serum creatinine(ummol/L)	58.00(52.00–67.00)	60.00(52.00–67.00)	0.585
Serum uric acid(ummol/L)	274.00(226.00-323.50)	303.50(229.00-361.50)	0.096
Urine protein+(%)	7.86%	5.56%	0.494
Urine blood+(%)	2.70%	2.78%	1.00
IgG(g/L)	13.10(10.40-16.66)	9.93(7.19–15.23)	<0.001*
IgA(g/L)	2.68(1.83–3.78)	2.09(1.19–3.09)	0.001*
IgM(g/L)	1.14(0.77–1.72)	0.84(0.58–1.39)	0.002*

Note: The Mann-Whitney U test was used for assessing the differences in the quantitative variables. The chi-squared test or Fisher’s exact test was used for assessing the differences in the categorical variables. * *P*<0.05

**Table 3:** Differences in indicators related to immune system between patients with and without oral candidiasis

	Patients without oral candidiasis	Patients with oral candidiasis	*P*
Total numbers of T cells + B cells + NK cells(cells/uL)	1527.70(1089.73-2000.85)	1324.84(834.42-1781.11)	0.021*
Numbers of T cells(cells/uL)	1106.76(773.08-1429.88)	930.74(558.93-1319.81)	0.018*
Percentages of T cells	72.62%(65.68-78.24%)	70.93%(63.46-76.78%)	0.219
Numbers of B cells(cells/uL)	176.16(116.20-275.82)	152.05(87.06-270.65)	0.110
Percentages of B cells	12.41%(9.16-17.59%)	12.32%(7.96-17.91%)	0.817
Numbers of Th cells (cells/uL)	617.99(427.21-857.28)	501.83(334.66-718.42)	0.005*
Percentages of Th cells	40.66%±9.38%	38.10%±11.51%	0.077
Numbers of Ts cells (cells/uL)	407.93(276.72-584.53)	401.63(226.51-603.53)	0.538
Percentages of Ts cells	27.99%(21.21-33.88%)	28.06%(19.35-37.00%)	0.563
Numbers of NK cells (cells/uL)	155.54(90.74-256.94)	146.64(82.17-262.91)	0.790
Percentages of NK cells	10.65%(7.06-17.00%)	12.97%(7.00-19.63%)	0.228
Numbers of Th1 cells (cells/uL)	103.46(45.55-188.66)	84.80(39.09-1157.68)	0.327
Percentages of Th1 cells	20.78%(12.87-30.00%)	19.10%(12.04-28.28%)	0.521
Numbers of Th2 cells (cells/uL)	4.82(2.71–7.67)	4.44(2.15–7.82)	0.422
Percentages of Th2 cells	0.78%(0.60-1.07%)	0.90%(0.60-1.25%)	0.165
Numbers of Th17 cells (cells/uL)	5.75(3.35–10.44)	5.23(2.91–8.81)	0.143
Percentages of Th17 cells	1.04%(0.62-1.63%)	1.10%(0.66-1.65%)	0.852
Numbers of Treg cells (cells/uL)	21.80(14.38–32.99)	21.60(10.92–33.01)	0.478
Percentages of Treg cells	3.72%(2.70-4.90%)	4.00%(2.82-5.81%)	0.143
Th cells/Ts cells	1.49(1.05–2.05)	1.36(0.88-2.00)	0.316
Th1 cells/Th2 cells	24.73(14.93–40.48)	24.12(12.18–38.89)	0.403
Th17 cells/Treg cells	0.28(0.16–0.49)	0.26(0.16–0.38)	0.356
Th1 cells/Treg cells	5.19(3.15–9.54)	4.80(2.52–8.35)	0.328
Th2 cells/Treg cells	0.26(0.16–0.42)	0.23(0.14–0.40)	0.414
B cells/Treg cells	7.43(4.72–11.99)	7.54(4.17–14.87)	0.942
NK cells/Treg cells	6.48(3.59–11.78)	5.97(3.16–13.31)	0.805
IL-2(pg/mL)	3.13(2.05–5.04)	2.99(2.04–6.99)	0.687
IL-4(pg/mL)	3.35(1.87–5.90)	2.82(1.93–6.33)	0.930
IL-6(pg/mL)	9.70(5.75–20.69)	13.79(5.49–39.74)	0.127
IL-10(pg/mL)	6.48(4.04–9.82)	7.01(4.83–11.36)	0.133
INF-r(pg/mL)	5.61(3.37–10.74)	6.48(3.47–16.08)	0.354
TNF-α(pg/mL)	4.49(2.40–8.92)	3.92(2.14–13.19)	0.747
Dosage of glucocorticoid (mg)	10(0–20)	15(4–20)	0.470
Immunosuppressant treatment(%)	75.43%	59.72%	0.006*

Note: When assessing the differences in the quantitative variables, if data were normally distributed, the T test was used, otherwise the Mann-Whitney U test was used .The chi-squared test or Fisher’s exact test were used for assessing the differences in the categorical variables. *P<0.05

**Table 4:** Differences in immunosuppressant treatment between patients with and without oral candidiasis

	Patients without oral candidiasis	Patients with oral candidiasis	χ²	*P*
Rapamycin(%)	93.53%	6.47%		
Hydroxychloroquine(%)	82.9%	17.10%	6.045	0.014*
Total Glucosides of Paeony Capsules(%)	76.00%	24.00%	7.832	0.005*

* There was a statistical difference in comparison to Rapamycin, *P<0.017

There were no significant differences in other clinical features (length of stay in hospital, haemoglobin, lymphocyte, platelets, alanine transaminase, aspartate aminotransferase, total protein, serum urea, serum creatinine, serum uric acid, urine protein, urine blood, the number of T cells, percentages of T cells, the number of B cells, percentages of B cells, percentages of Th cells, the number of NK cells, percentages of NK cells, the number of Th1 cells, percentages of Th1 cells, the number of Th2 cells, percentages of Th2 cells, the number of Th17 cells, percentages of Th17 cells, the number of Treg cells, percentages of Treg cells, Th cells versus Ts cells, Th1 cells versus Th2 cells, Th17 cells versus Treg cells values, Th2 cells versus Treg cells values, B cells versus Treg cells values, NK cells versus Treg cells values, IL-2, IL-4, IL-6, IL-10, INF-r, TNF-α, and the dosage of glucocorticoid) between the patients with oral candidiasis and those without oral candidiasis (Tables 2 and 3, *P* > 0.05).

### Risk factors related to oral candidiasis

Preliminary research was carried out using multivariate logistic regression to investigate the possible independent risk variables associated with oral candidiasis in individuals with Sjogren’s syndrome. As shown in Tables 4 and 5, male sex (OR:1.02, 95%CI:0.99 − 0.47, *P* = 0.002), UWS (OR:0.004, 95%CI:0.00-0.47, *P* = 0.023), greater white blood cell counts (OR:1.22, 95%CI:1.07–1.40, *P* < 0.001), lower haemoglobin levels (OR:0.97, 95%CI:0.95–0.99, *P* = 0.007), lower serum albumin levels (OR:0.88, 95%CI:0.81–0.94, *P* < 0.001), lower IgG levels (OR:0.91, 95%CI:0.85–0.98, *P* = 0.011), lower IgA levels (OR:0.75, 95%CI:0.57–0.98, *P* = 0.011), lower IgM levels (OR:0.91, 95%CI:0.36–0.90, *P* = 0.015), greater percentages of Treg cells (OR:1.29, 95%CI:1.05–1.59, *P* = 0.015), lower Th1 versus Treg cells values (OR:1.14, 95%CI:1.04–1.24, *P* = 0.004), and without immunosuppressant treatment might be independent risk factors (OR:0.32, 95%CI:0.15–0.69, *P* = 0.011) for oral candidiasis infection in Sjogren’s syndrome patients. The preliminary results must be confirmed in a large sample of patients in the future.

**Table 5:** Analysis of risk factors related to oral candidiasis infection in Sjogren’s syndrome patients

Variables	β	*P*	OR	OR 95%CI
Male	2.01	0.002*	7.43	2.07–26.63
Basal salivary flow rate	-5.55	0.023*	0.00	0.00-0.47
White blood cells	0.20	<0.001*	1.22	1.07–1.40
Hemoglobin	-0.03	0.007*	0.97	0.95–0.99
Serum albumin	-0.13	<0.001*	0.88	0.81–0.94
IgG	-0.09	0.011*	0.91	0.85–0.98
IgA	-0.29	0.038*	0.75	0.57–0.98
IgM	-0.537	0.015*	0.57	0.36–0.90
Percentages of Treg cells	0.26	0.015*	1.29	1.05–1.59
Th1 cells/Treg cells	0.13	0.004*	1.14	1.04–1.24
Immunosuppressant treatment	-1.15	0.004*	0.32	0.15–0.69

Note: The risk factors of oral candidiasis were screened by multi-factor Logistic regression analysis. *P<0.05

## Discussion

Candidiasis is the most common fungal infection of the oral cavity. The pathogenesis of this infection is unknown, but several systemic (e.g., immunosuppression or endocrine disruption) and local factors (e.g., decreased salivary flow, use of dentures, high-sugar diets) have been linked to an overgrowth of *Candida* species, with *Candida* albicans being the most commonly linked species with oral lesions [6, 19]. In this study, 479 patients with Sjögren’s syndrome (SS) were included and categorised into two groups: patients with oral candidiasis and patients without oral candidiasis. A comprehensive comparison of the clinical features between the two groups was conducted. Our analysis revealed a notable disparity in the age of patients between the oral candidiasis group and the nonoral candidiasis group, with the former having a substantially greater average age. Additionally, there was a significantly greater percentage of male patients in the oral candidiasis group than in the nonoral candidiasis group. This might be attributed to the high incidence of smoking and inadequate oral hygiene among male patients. While the male-to-female ratio of SS patients ranged from 1:9 to 1:10, it was observed that older male SS patients had a greater likelihood of developing oral *Candida* infections.

SS is a complex autoimmune, chronic, and systemic illness marked by lymphocyte infiltration and eventual death of the exocrine glands. It typically affects the salivary and lacrimal glands [22]. According to previous studies, 88% of SS patients have decreased salivary flow, and 75–92% of patients have dry mouth [23]. Saliva possesses mechanical, antibacterial, and antifungal properties that help to preserve the mouth mucosa. Saliva includes antimicrobial defence mechanisms, such as IgA, lysozyme, and lactoferrin [24, 25]. Therefore, hyposalivation not only alters the volume of saliva but also alters the oral microbiota, increasing the risk of opportunistic fungal infections, including *Candida* infections [14, 26]. *Candida* adherence to epithelial and acrylic surfaces is also improved at low pH and flow rates [27]. In our study, we examined the baseline salivary flow rate and poststimulation salivary flow rate in patients in the SS oral *Candida* infection and noninfected groups. We found that the UWS and SWS were negatively correlated with oral *Candida* infection. Furthermore, logistic regression analysis demonstrated that individuals with oral *Candida* infection had decreased levels of UWS. We propose that decreased levels of UWS have greater significance than decreased levels of SWS in promoting oral *Candida* infections in patients with SS. This might be attributed to the fact that the UWS provides a more accurate reflection of the patient’s salivary flow levels for the most of the time. Serrano et al. reported that 87.5% of SS patients with clinical candidiasis presented reduced pH levels and salivary flow in both the UWS and SWS. A significant negative correlation was detected between the number of CFUs/mL of *Candida* albicans and the levels of UWS and SWS [8]. Hence, we recommend closely monitoring patients with low levels of UWS. The primary emphasis should be on enhancing oral hygiene practices and considering the use of topical preventive antifungal treatment to reduce the occurrence of oral *Candida* infections in individuals with SS.

The relationship between the oral microbiome and autoimmune disorders is complicated. In the case of SS, activated CD4^+^ T cells and B cells enter the salivary glands [28]. Salivary gland damage may change the composition of salivary proteins, resulting in oral dysbiosis. The oral microbiota may impact autoimmune illnesses via toll-like receptor activation, molecular mimicry, epitope dissemination, and antigenic persistence [29]. Studies in animal models have revealed that peptides from oral bacteria stimulate Ro60-reactive T cells [30], which then activate B cells as plasma cells that release SS antigen A [31]. *Candida* stimulates human Th17 cell responses and has a crucial physiological function in the progression of SS [32, 33]. Several studies have shown that *Candida* infection is an important environmental factor contributing to SS [34] and that individuals with a history of infection have an increased likelihood of developing SS [35]. Therefore, it is essential to thoroughly examine the correlation between *Candida* infection and SS. Our investigation revealed that individuals suffering from SS oral *Candida* infection had elevated blood leukocyte counts, decreased serum albumin levels, and reduced levels of the serum immunoglobulins IgG, IgA, and IgM. The overall counts of T cells, B cells, and NK cells, and of Th cells were notably lower in patients with SS than in those without oral candidiasis. These findings indicate a potential association between oral *Candida* infection and the autoimmune condition of the patient, suggesting that oral candidiasis may serve as an indicator of alterations in the whole-body immune response and the microenvironment. Decreased levels of serum immunoglobulin IgG, IgA, and IgM were identified as separate risk factors for oral *Candida* infection. Oral *Candida* infections are more prone to develop when the immune system is compromised, particularly in conjunction with decreased T cells and B cell levels. Hence, we propose that the management of SS in conjunction with oral candidiasis should include not only localised therapy of the oral cavity but also the modulation of the systemic immune system.

The current treatment for oral candidiasis is based on the application of topical and systemic antifungal medications. However, antifungal medications are associated with serious side effects, including kidney and liver damage, vomiting, nausea, and diarrhoea [36, 37]. Probiotics are emerging therapeutic and preventative agents for oral candidiasis. Li et al. reported that probiotic preparations derived from *Lactobacillus bulgaricus* and *Streptococcus thermophilus* combined with oral topical antifungal medicine (mycobacterial) were more successful than standard therapies for *Candida*-associated stomatitis [38]. Probiotics are useful for lowering the oral *Candida* burden in SS patients [39]. However, a small number of randomised controlled trials revealed little indication that probiotics lower the risk of *Candida* in persons with SS [40]. In this study, a low Treg percentage was found to be an independent risk factor for oral *Candida* infection. Treg cells exert immunosuppressive effects on both nonmicrobial and microbial antigens, hence mitigating tissue damage caused by inflammation. These effects are achieved via many suppressive mechanisms [41]. Additionally, our study revealed that compared with patients treated with hydroxychloroquine and total glucosides of peony capsules, patients treated with rapamycin had a lower rate of oral candidiasis infection. Rapamycin was found to increase the number of circulating Treg cells in liver transplant patients [42]. Rapamycin increases the abundance of Treg cells in individuals with rheumatoid arthritis, increasing the probability of achieving long-lasting, continuous clinical remission and reducing the possibility of disease relapse [43]. We hypothesise that the use of immunosuppressive medications may decrease the occurrence of oral *Candida* infections in individuals with SS by increasing the abundance of Treg cells. Additional future studies are necessary to determine the potential of rapamycin as a therapy for oral *Candida* infections in patients with SS.

In this study, we don’t discuss the categorization of different clinical types of oral candidiasis. This is one of the limitations of our study and we will improve it in our subsequent studies.

## Conclusions

In this population-based study, patients with oral candidiasis were older than those without oral candidiasis. Compared with female patients, male SS patients had a greater incidence of oral *Candida* infections. Individuals with low UWS and SWS are susceptible to oral *Candida* infection. The immunological state of the whole body is a significant factor in the development of oral *Candida* infection in people with SS. Rapamycin has the potential to decrease the occurrence of oral *Candida* infections in individuals with SS by increasing the proportion of Treg cells. However, more investigations on the underlying molecular mechanisms are needed.

### Electronic supplementary material

Below is the link to the electronic supplementary material.

Supplementary Material 1

Supplementary Material 2

## Data Availability

The datasets generated and/or analysed during the current study are not publicly available but are available from the corresponding author on reasonable request.

## Abbreviations

SS: Sjogren’s Syndrome
UWS: Unstimulated whole saliva
SWS: Stimulated whole saliva
