# Supplementary material for: Clinical features and risk factors for Sjogren’s syndrome patients suffering from oral candidiasis in Shanxi, China
Source: BMC Oral Health. 2024 Jul 17;24:812. doi: 10.1186/s12903-024-04595-x (PMC11256585; doi:10.1186/s12903-024-04595-x)
Supplement: Supplementary file 1 — Supplementary Material 1 [file 12903_2024_4595_MOESM1_ESM.docx]

**Supplementary Table 1.** Analysis of risk factors related to oral candidiasis infection in Sjogren's syndrome patients.

| Variables | β | P | OR | OR 95%CI |
| --- | --- | --- | --- | --- |
| Age | 0.02 | 0.253 | 1.02 | 0.99-1.06 |
| Length of stay in hospital | 0.01 | 0.856 | 1.01 | 0.94-1.08 |
| Stimulated salivary flow rate | -0.31 | 0.387 | 0.73 | 0.37-1.48 |
| Lymphocyte | -0.22 | 0.471 | 0.80 | 0.45-1.45 |
| Platelets | 0.00 | 0.758 | 1.00 | 1.00-1.01 |
| Alanine transaminase | 0.01 | 0.645 | 1.01 | 0.98-1.04 |
| Aspertate aminotransferase | -0.01 | 0.515 | 0.99 | 0.95-1.03 |
| Total protein | 0.05 | 0.052 | 1.06 | 1.00-1.12 |
| Serum urea | -0.18 | 0.072 | 0.84 | 0.69- 1.02 |
| Serum creatinine | 0.00 | 0.735 | 1.00 | 0.98-1.03 |
| Serum uric acid | 0.00 | 0.195 | 1.00 | 1.00-1.01 |
| Urine blood+ | -1.24 | 0.107 | 0.29 | 0.06-1.31 |
| Urine protein+ | -0.64 | 0.561 | 0.53 | 0.06-4.54 |
| Total numbers of T cells+B cells+NK cells | 0.00 | 0.65 | 1.00 | 0.99-1.01 |
| Numbers of T cells | 0.00 | 0.649 | 1.00 | 0.99-1.01 |
| Percentages of T cells | 0.01 | 0.068 | 1.01 | 1.00-1.02 |
| Numbers of B cells | 0.01 | 0.285 | 1.01 | 1.00-1.02 |
| Percentages of B cells | -0.01 | 0.819 | 0.99 | 0.94-1.05 |
| Numbers of Th cells | 0.00 | 0.848 | 1.00 | 0.99-1.01 |
| Percentages of Th cells | 0.02 | 0.575 | 1.02 | 0.94-1.11 |
| Numbers of Ts cells | 0.00 | 0.747 | 1.00 | 0.99-1.01 |
| Percentages of Ts cells | 0.00 | 0.959 | 1.00 | 0.99-1.00 |
| Numbers of NK cells | 0.00 | 0.488 | 1.00 | 0.99-1.01 |
| Percentages of NK cells | 0.01 | 0.791 | 1.01 | 0.92-1.12 |
| Numbers of Th1 cells | 0.00 | 0.974 | 1.00 | 0.99-1.00 |
| Percentages of Th1 cells | -0.04 | 0.090 | 0.96 | 0.92-1.01 |
| Numbers of Th2 cells | 0.04 | 0.352 | 1.04 | 0.96-1.13 |
| Percentages of Th2 cells | 0.41 | 0.374 | 1.50 | 0.61-3.67 |
| Numbers of Th17 cells | 0.02 | 0.732 | 1.02 | 0.92-1.13 |
| Percentages of Th17 cells | 0.05 | 0.892 | 1.05 | 0.50-2.21 |
| Numbers of Treg cells | 0.00 | 0.839 | 1.00 | 0.99-1.01 |
| Percentages of Treg cells | 0.26 | 0.015 | 1.29 | 1.05-1.59 |
| Th cells/Ts cells | -0.01 | 0.660 | 0.99 | 0.95-1.03 |
| Th1 cells/Th2 cells | -0.01 | 0.427 | 0.99 | 0.97-1.01 |
| Th17 cells/Treg cells | -1.09 | 0.362 | 0.34 | 0.03-3.48 |
| Th2 cells/Treg cells | -0.34 | 0.461 | 0.71 | 0.28-1.77 |
| B cells/Treg cells | 0.00 | 0.923 | 1.00 | 0.98-1.02 |
| NK cells/Treg cells | 0.00 | 0.905 | 1.00 | 0.98-1.02 |
| IL-2 | 0.01 | 0.822 | 1.01 | 0.91-1.13 |
| IL-4 | -0.01 | 0.620 | 0.99 | 0.96-1.02 |
| IL-6 | 0.00 | 0.476 | 1.00 | 1.00-1.01 |
| IL-10 | 0.00 | 0.864 | 1.00 | 0.96-1.03 |
| INF-γ | -0.01 | 0.450 | 0.99 | 0.99-1.04 |
| TNF-α | 0.02 | 0.200 | 1.02 | 0.99-1.04 |
| Dosage of glucocorticoid | -0.00 | 0.534 | 0.99 | 0.96-1.02 |

Note:The risk factors of oral candidiasis were screened by multi-factor Logistic regression analysis. **P*＜0.05. IL-2, interleukin-2; IL-4, interleukin-4; IL-6, interleukin-6; IL-10,interleukin-10; INF-γ, interferon-γ；TNF-α,tumor necrosis factor-α.
