# Supplementary figures and images for: Clinical features and risk factors for Sjogren’s syndrome patients suffering from oral candidiasis in Shanxi, China
Source: BMC Oral Health. 2024 Jul 17;24:812. doi: 10.1186/s12903-024-04595-x (PMC11256585; doi:10.1186/s12903-024-04595-x)

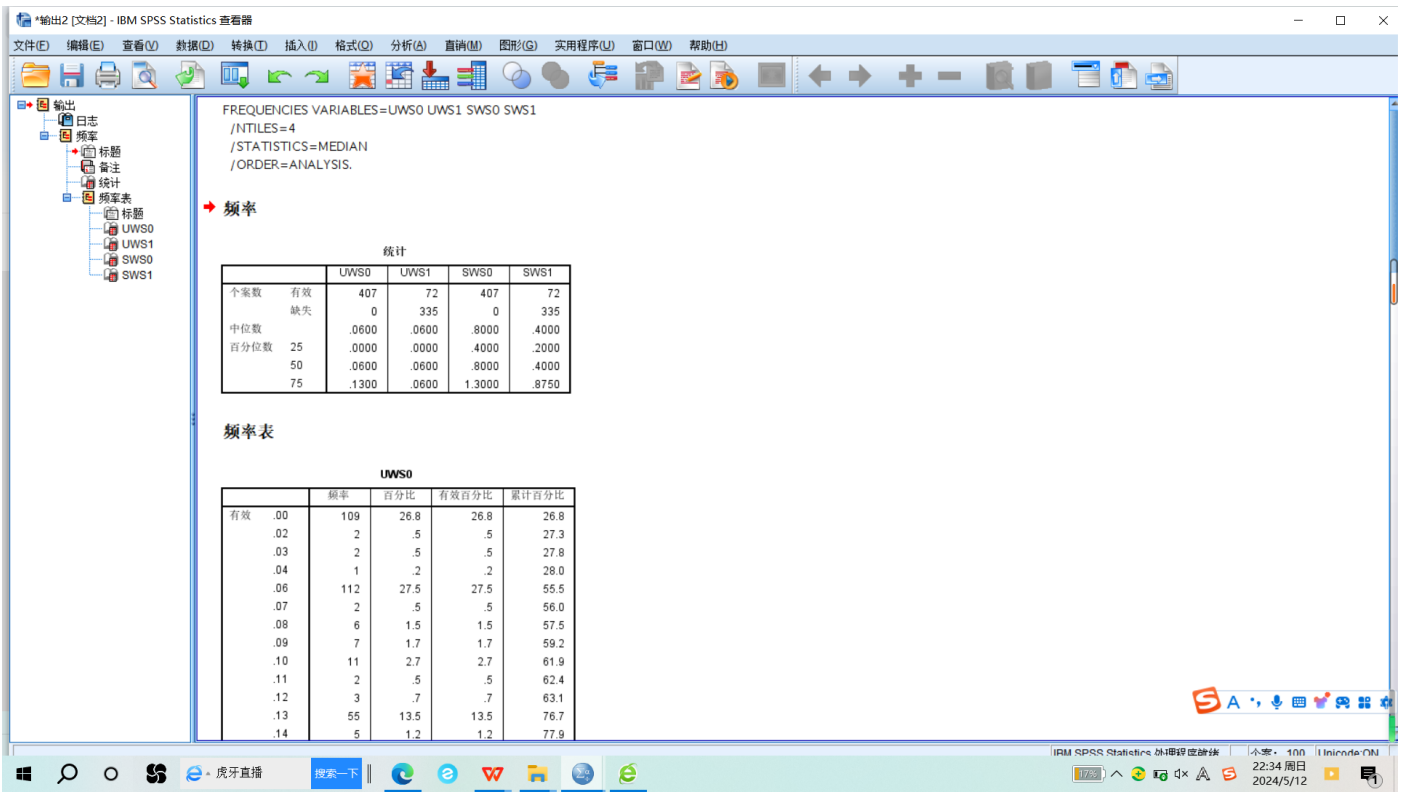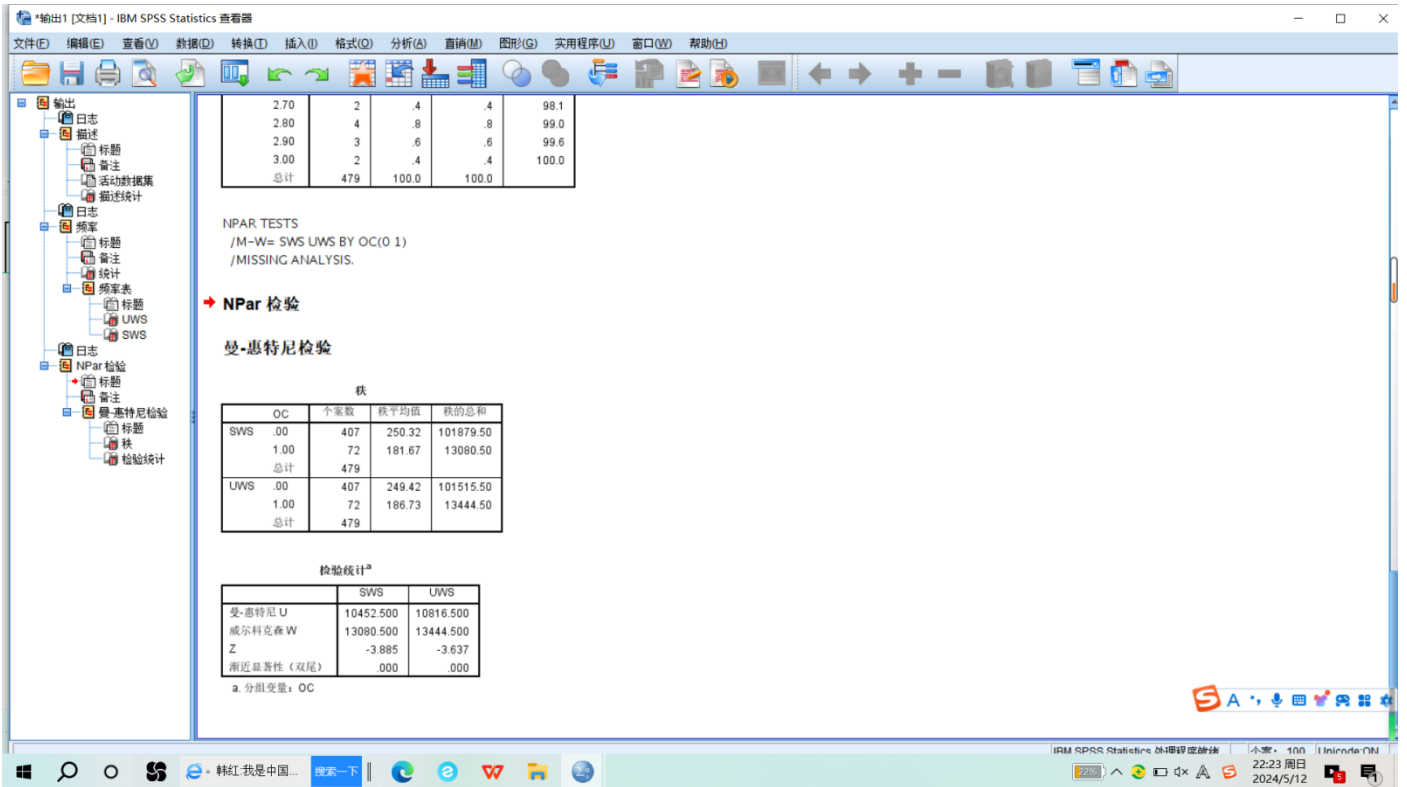

Supplement: Supplementary file 2 — Supplementary Material 2 [file 12903_2024_4595_MOESM2_ESM.pdf]
